# Characterization of the *Burkholderia cenocepacia TonB* Mutant as a Potential Live Attenuated Vaccine

**DOI:** 10.3390/vaccines5040033

**Published:** 2017-09-28

**Authors:** Gonzalo A. Pradenas, Julia N. Myers, Alfredo G. Torres

**Affiliations:** 1 Department of Microbiology and Immunology, University of Texas Medical Branch, Galveston, TX 77555, USA; gonzalo.pradenas@gmail.com (G.A.P.); jnmyers@utmb.edu (J.N.M.); 2 Sealy Center for Vaccine Development, University of Texas Medical Branch, Galveston, TX 77555, USA

**Keywords:** *Burkholderia cenocepacia*, *tonB*, attenuated strain, vaccine

## Abstract

*Burkholderia cenocepacia* is an opportunistic pathogen prevalent in cystic fibrosis patients, which is particularly difficult to treat, causing chronic and eventually fatal infections. The lack of effective treatment options makes evident the need to develop alternative therapeutic or prophylactic approaches. Vaccines, and live attenuated vaccines, are an unexplored avenue to treat *B. cenocepacia* infections. Here we constructed and characterized a *B. cenocepacia tonB* mutant strain, which was unable to actively transport iron, to test whether this single gene deletion mutant (strain renamed GAP001) protected against an acute respiratory *B. cenocepacia* lethal infection. Here we show that the mutant strain GAP001 is attenuated, and effective at protecting against *B. cenocepacia* challenge. Intranasal administration of GAP001 to BALB/c mice resulted in almost complete survival with high degree of bacterial clearance.

## 1. Introduction

The *Burkholderia* genus encompasses several environmental, Gram-negative aerobes, ordinarily found in soil and ground water [1]. Within this genus, a group of 20 heterogeneous species could be found, which are classified as the *Burkholderia cepacia* complex (Bcc) subgroup. These bacteria are known to be opportunistic pathogens and causative agents of respiratory infections in patients with cystic fibrosis (CF) and chronic granulomatous disease (CGD) [1,2,3,4]. In CF patients, Bcc infections might pose a mortal risk, since they can progress and cause a rapid deterioration in lung function [5], reaching in some cases, a fatal acute bacteremia with necrotizing pneumonia outcome, known as the “cepacia syndrome” [6].

Bcc is rarely successfully eradicated from patients, since the infection presents with several characteristics that make it difficult to treat. Among them, were found high and wide antibiotic resistance, antimicrobial peptide resistance, biofilm production, and the ability to enter and survive intracellularly within respiratory epithelial cells and macrophages [6,7,8].

Different approaches have been investigated to find an effective treatment. Among them, several therapeutic approaches have been proposed and the search for prophylactic vaccines has also been investigated, as a way to provide protection instead of combating infection eradication [4,9].

There are a few in vivo mouse vaccination studies that have reported different degrees of protection against Bcc [10,11,12]. The most recent studies used purified recombinant proteins, Linocin and OmpW, which elicited mixed Th1/Th2/Th17 serological responses [11], or whole outer membrane protein preparations which, depending on the formulation, were capable of both providing immunity and a reduced *B. multivorans* burden or resulted in nearly sterilizing immunity against *B. cenocepacia* and *B. multivorans* [12].

Other vaccine types, such as live attenuated or heat-killed whole-cell-based vaccines have not been evaluated in the case of Bcc [4]. In contrast, live attenuated vaccines have been tested with some success in mice models, against related species such as *B. mallei* or *B. pseudomallei* [13]. An attenuated vaccine for *B. mallei* presented survival rates of between 75 to 100% when tested in acute inhalational models [14]. In this study, the attenuation was generated by disruption of the *tonB* gene, encoding the energizer protein that activates iron transport through the outer membrane. Since the mechanisms of acquisition of iron compounds are closely linked to the pathogenic process in several bacterial species, they have been tested as a potential target for vaccine development. Scavenging iron from the host by bacteria using siderophores and/or high-affinity outer-membrane receptors is particularly relevant, because the host environment has limited amounts of free iron [15]. Similar issues might be important for long term Bcc infections in CF patients, because it has been shown that *B. cenocepacia* upregulates iron-uptake-related genes, including those encoding siderophore biosynthesis, compared to the initial stages of the same infection [16].

Given that *Burkholderia* iron uptake systems have some degree of redundancy [17,18], targeting the TonB protein—the essential membrane energy protein interacting with all outer membrane receptors that are involved in iron uptake—is a plausible approach for attenuation, as demonstrated in different bacterial species [14,19,20]. Further, it has been demonstrated for *Klebsiella pneumoniae* and *B. mallei*, that this attenuated mutation can confer protection against challenge with their corresponding wild-type counterparts [14,20,21]. Therefore, in this study we constructed and characterized a *B. cenocepacia tonB* mutant and evaluated its role as live attenuated vaccine, conferring protection against *B. cenocepacia* K56-2 using an acute murine infection model.

## 2. Material and Methods

### 2.1. Bacterial Strains and Growth Conditions

The bacterial strains used in this study are listed in Table 1. For liquid cultures, two to three colonies were inoculated into 20 mL of LB broth. Liquid cultures were then incubated overnight (18 h) at 37 °C with agitation (200 rpm). Challenge and vaccination doses were prepared from overnight (12 h) LB cultures and the used dose was resuspended in phosphate-buffered saline (PBS).

### 2.2. Growth Kinetics Assay

Overnight cultures were used to inoculate 30 mL of LB with 8 × 10^6^ CFU (Colony Forming Units) of each strain. The cultures were then incubated with agitation at 37 °C. At the indicated time points, the OD_600_ was measured. 

### 2.3. DNA, PCR and Cloning Methods

Cloning methods were performed as previously described [14]. Chromosomal DNA was isolated by using the DNeasy Qiagen blood and tissue kit (Qiagen, Germantown, MD, USA) and plasmid DNA was isolated using the QIAGEN Plasmid mini kit. PCR was performed with Phusion High-Fidelity DNA polymerase from New England Biolabs (NEB, Ipswich, MA, USA), using the following PCR protocols: For tet^R^—1 amplification, a cycle of 98 °C for 1 min, 29 cycles of 98 °C for 15 s, 50 °C for 30 s, 72 °C for 85 s, and 1 cycle of 72 °C for 10 min. For *tonB*—1 amplification, a cycle of 98 °C for 1 min, 29 cycles of 98 °C for 15 s, 60 °C for 60 s, 69 °C for 60 s, and 1 cycle of 69 °C for 10 min. The amplicons were isolated with the QIAquick PCR purification kit or extracted from agarose gel with the QIAquick gel extraction kit. The restriction enzymes, Gibson assembly kit and T4 DNA ligase were obtained from NEB, and used according to the providers’ instructions. The primers used in this work were purchased from Integrated DNA Technologies (Coralville, IA, USA).

### 2.4. pGPTCR Plasmid Construction

The kanamycin resistance cassette from pMO130 was excised using SpeI and XbaI restriction enzymes, and replaced by the tetracycline resistance (Tet^R^) cassette obtained from pACYC184. The Tet^R^ amplicon was obtained using the following set of primers: Tet forward (ACC AAA ACG ATC TCA AGA); Tet reverse (CCG GCG ACT AGT GGT GCC GGC TTC CA) (the underlined sequence indicates the SpeI site), the amplicon was purified and then digested with XbaI and SpeI (the amplicon possesses an internal XbaI site). The resulting fragment was gel separated and purified and ligated to the pMO130 (without cassette), creating the pGPTcR plasmid.

### 2.5. Construction of the GAP001 Strain

Matched adaptamers specifically designed for Gibson assembly were amplified via touchdown PCR. The primers sequences were as follows: Gibson Δ*tonB* Up forward (CCG CCC TGC AGC GGA TCC CTC TAG AAC CGG TGT TCA TGG CCA); Gibson Δ*tonB* Up reverse (CGT GGC TCC ATA ACC TGC CTC ATC CGG AAG); Gibson Δ*tonB* Dw forward (AGG CAG GTT ATG GAG CCA CGC GGC CGT C); and Gibson Δ*tonB* Dw reverse (GAG ATA AAT TGC ACT GAA ATC TAG ACC CAG GCT TGT CAA CAC CCG). The adaptamers and an XbaI cut pGPTcR were fused together using a Gibson Assembly mastermix (NEB) according manufacturer instructions, creating a pGPTcR-tonB plasmid. The segment contains a 1507 bp chimeric fragment encompassing the flanking regions plus the first 43 codons of the *tonB* gene. The pGPTcR-tonB plasmid was chemically transformed into *E*. *coli* S17-1 and then introduced into *B*. *cenocepacia* through bi-parental mating. Merodiploids candidates were obtained by Tet and polymyxin B (Pb^r^) agar plate selection, as well from the detection of the *xylE* reporter gene product by the presence of a color shift (to yellow) after exposure to pyrocatechol.

Single deletion mutants were selected, by duplicate plate, on YT agar supplemented with 200 μM FeSO_4_, with and without 20% sucrose. The obtained candidates were tested for Tet susceptibility and the *tonB* deletion was checked via PCR and sequencing confirmation using primers, forward (TGC GCG ATA CCG TAG CTT) and reverse (GGT CCG GCG TAA AAA AC). The confirmed mutant strain was named GAP001.

### 2.6. Iron Utilization Assay

Overnight cultures were diluted to 1 × 10^5^ CFU/mL in LB + 50 or 300 μM of 2,2’-dipyridyl (for GAP001 and K56-2, respectively) and poured onto plates, as previously described [23]. The iron sources were spotted on the surface of the LB plates, which were incubated at 37 °C for 48 h. 10 μL of the following compounds at the specified concentrations were used: hemin, 8.0 μM; hemoglobin, 4.5 μM; myoglobin, 4.5 μM; transferrin, and lactoferrin, both at 30 μM or FeSO_4_, 10 mM. Iron utilization was determined as bacterial growth around the spotted area.

### 2.7. Siderophore Secretion Assay

Ten μL samples of overnight cultures, grown in LB or LB + 200 μM FeSO_4_, were spotted onto CAS (Chrome Azurol S) agar plates and incubated at 37 °C. Halos were then monitored and the diameter of the color change was measured over 48 h. CAS agar plates was prepared as described [24]. An unpaired *t*-test was performed (*p* ≤ 0.05) between the strain-specific halos produced.

### 2.8. In Vitro Survival Assays

Cellular uptake and survival assays were performed as previously described [25]. Each assay was performed in quadruplicate. Murine macrophage cell line RAW 264.7 (ATCC^®^ TIB-71^™^) were incubated at 37 °C with 5% CO_2_ in 24-well plates (Corning Inc., Corning, NY, USA) at a concentration of 5 × 10^5^ cells per well. Cells were grown in DMEM complete medium and *B. cenocepacia* suspensions were added to cells at a multiplicity of infection (MOI) of 10, followed by centrifugation at 250× *g* for 5 min and incubation in 37 °C with 5% CO_2_ for up to 8 h. Two hours post-infection (hpi), the monolayers were washed twice with sterile Dulbecco’s phosphate-buffered saline (DPBS) (Cellgro, Tewksbury, MA, USA) and the media replaced with DMEM complete media supplemented with 250 μg/mL gentamicin and 500 μg/mL of ceftazidime. Monolayers were lysed with 0.1% Triton X-100 in PBS at the indicated times, serial dilutions plated and incubated at 37 °C for 24–48 h. The percentage of invasion was calculated as: (uptake CFU/ total inoculum CFU) × 100.

### 2.9. In Vivo Survival Study

Anesthetized BALB/c mice (n = 5 per treatment) were inoculated intranasally (i.n.) with 5 × 10^7^ CFU of GAP001 and *B. cenocepacia* K56-2. Bacteria were resuspended in phosphate-buffered saline (PBS) in a total volume of 50 μL (25 μL/naris). Mice were monitored and deaths recorded over a period of six days. Survival curves were generated and analyzed by using the Kaplan–Meier method. Significant differences (*p* ≤ 0.05) were determined via a log rank test.

This study was carried out in strict accordance with the recommendations in the Guide for the Care and Use of Laboratory Animals of the National Institutes of Health. The protocol was approved by the Animal Care and Use Committee of the University of Texas Medical Branch (Protocol Number 0503014D).

### 2.10. Vaccine Study

Anesthetized BALB/c mice (n = 8 per treatment) were given a series of two i.n. immunizations, at two week intervals, with PBS or 5 × 10^7^ CFU of GAP001 resuspended in PBS in a total volume of 50 μL (25 μL/ naris). Mice were challenged 14 days after the second boost with 5 × 10^7^ CFU of *B*. *cenocepacia* K56-2 Nac^R^, diluted in PBS in 50 μL (25 μL/ naris). Mice were monitored and deaths were recorded until the end of the study. Survival curves were generated and analyzed using the Kaplan–Meier method. Significant differences (*p* ≤ 0.0005) were ascertained via a log rank test.

### 2.11. Specific Immunoglobulin Analysis

Serum extracted from PBS or GAP001-vaccinated BALB/c mice at 10 days after the second boost were evaluated for *B. cenocepacia*-specific IgG1, IgG2a and IgG antibodies using an ELISA performed in 96-well Costar high-binding microplates (Corning Inc., Corning, NY, USA). Briefly, 12 h cultures of *B. cenocepacia* K56-2 Nac^R^ were pelleted and resuspended in PBS, then inactivated for 25 min at 60 °C and diluted to a concentration of 10 μg/mL in PBS; the wells were coated with 100 μL/well of diluted suspension and incubated overnight at 4 °C. Wells were washed twice with wash buffer (0.05% Tween 20 in PBS) and incubated with 250 μL of blocking solution (1% bovine serum albumin, 0.05% Tween 20 in PBS) for2h at room temperature (RT). After blocking, plates were washed twice with wash buffer. Mouse sera was serial diluted (two-fold) in triplicate with sample diluent (PBS, 0.5% bovine serum albumin, and 0.05% Tween 20 in PBS), then 100 μL volume of diluted sera was added to sample wells along with 100 μL of 1:500 anti IgG or 5000 anti-Ig subclasses conjugated to horseradish peroxidase (Southern Biotechnology Associates, Inc., Birmingham, AL, USA). These were incubated at RT for 3 h. The plates were washed four times with wash buffer and then 100 μL of tetramethylbenzidine (TMB) substrate solution (eBioscience, Inc., San Diego, CA, USA) were added. After incubating for 15 min, 100 μL of stop solution (2N H_2_SO_4_) was added and the wells were read at 450 nm using an Epoch microplate spectrophotometer (BioTek Instruments, Inc., Winooski, VT, USA). The results were reported as the reciprocal of the highest titer giving an optical density (OD) reading higher than twice the background +1 standard deviation (SD). All assays were performed in triplicate, and results were reported as the mean reciprocal endpoint titer ± SD.

## 3. Results

### 3.1. tonB Mutant Generation

The first step to generate a *B. cenocepacia* deletion mutant via allelic exchange was to modify the pMO130 plasmid allowing the use of tetracycline resistance as a selection marker. The kanamycin resistance cassette present in pMO130 was excised, and replaced with a tetracycline cassette resulting in the pGPTcR. The construct was transformed in *E. coli* and screened for tetracycline resistance. The plasmids were isolated from positive candidates, and the presence of the tetracycline resistance cassette was verified by PCR and restriction analysis.

The pGPTcR was used to generate a *tonB*-deficient *B. cenocepacia* by allelic exchange, as an unmarked deletion mutant, as previously described for *B. mallei* [26], the resulting *tonB* deletion mutant was named GAP001. Similar to the *B. mallei tonB* [14], GAP001 appears as yellow-colored colonies, a phenotype thought to be associated with siderophore secretion [14,27].

### 3.2. GAP001 Iron Utilization and Growth Characterization

**Figure 1:**
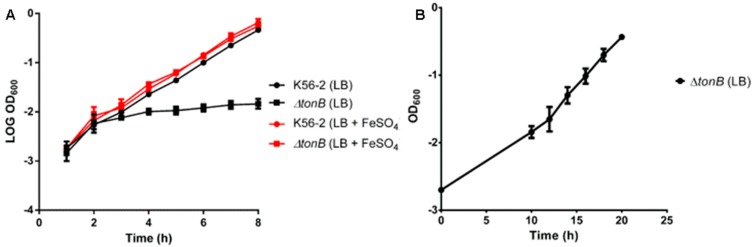
GAP001 impaired growth is rescued by iron supplementation. Overnight cultures of wild type and GAP001 were diluted 1:100 in 30 mL of LB broth (black circle and black square) or 30 mL of LBG + 200 µM FeSO_4_ (red circle and red square), OD_600_ was determined every hour. Growth curves up to 8 h (**A**). GAP001 growth curve up to 20 h (**B**).

The *tonB* mutant strain GAP001 was characterized at several levels. First the growth rate was determined in LB media with and without 200 µM FeSO_4_. GAP001 showed adeficient growth in LB media alone (Figure 1A,B), compared with the wild type strain. However, when the media was supplemented with free iron, the growth rate of the GAP001 strain was indistinguishable from the wild type strain (Figure 1A).

One classic phenotype of the *Burkholderia tonB* mutants is an increased siderophore production [14] as the bacteria tries to compensate the hindered iron deficiency with increased iron capture and potential uptake. This siderophore release was determined using a CAS agar assay because the siderophores are able to sequester the iron present in the CAS media, generating media discoloration around the growing bacteria. Siderophore secretion was measured after at 48 h and expressed as the area around the halo (Table 2). GAP001 produced significantly larger halos (41.0 ± 1.26 mm) compared to the wild type (22.0 ± 0.36 mm), both consistent with previous reports [14].

Given that TonB energization of the iron receptors is required for the active uptake of different iron sources, the ability of the *tonB* mutant to take up free and bound iron was determined. The iron utilization assay was performed with different sources of iron: FeSO_4_, hemoglobin, hemin, lactoferrin, and transferrin. Iron source utilization was measured as noticeable bacterial growth around the disk containing the iron source. The *B. cenocepacia* wild-type strain was able to grow by utilizing all iron sources, while GAP001 was only capable of utilizing FeSO_4_ (Table 3).

### 3.3. GAP001 Intracellular Uptake and Survival Characterization

**Figure 2:**
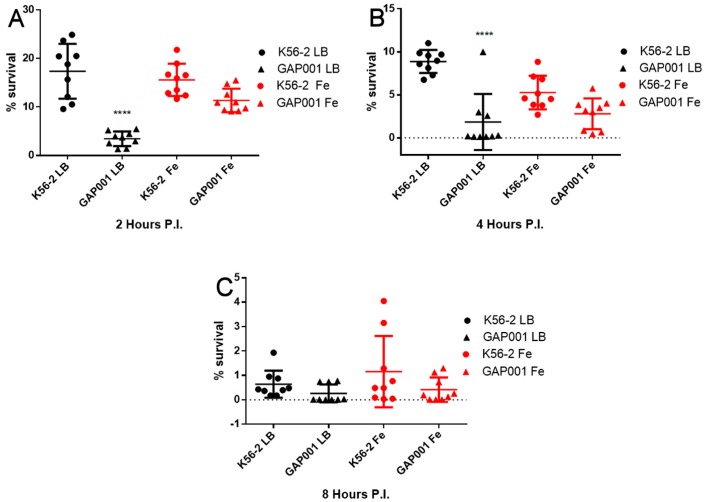
GAP001 intracellular uptake and survival in RAW264.7 cells. Cells were infected with *B. cenocepacia* or GAP001 grown either in LB alone or LB with 200 μM FeSO_4_, at a multiplicity of infection (MOI) of 10. At 2 (**A**), 4 (**B**) and 8 (**C**) h, cells were lysed and bacterial uptake and survival was determined by dilution plating. Individual data points are shown as well as mean ± S.D. Levels of significance (*p* < 0.0001).

To further characterize GAP001, *in vitro* intracellular uptake and survival was determined using RAW 264.7 murine macrophages. At the beginning of the infection, when grown in LB alone, uptake of the *tonB* mutant was significantly lower compared with the wild-type strain (*p <* 0.001, Dunnett’s test) at 2 hpi (Figure 2A). As expected, the GAP001 strain grown in LB supplemented with 200 μM FeSO_4_ did not show significant differences to the wild-type strain but was different to the GAP001 grown in LB alone. At 4 hpi, the survival numbers were reduced for all the strains and conditions, particularly the GAP001 stain, which showed similar behavior in LB with and without FeSO_4_ and as compared to the wildtype strain (Figure 2B). After 8 hpi, both the GAP001 and *B. cenocepacia* K56-2 survival rate inside raw macrophages diminished to 1% or lower, without significant differences in any condition tested.

### 3.4. GAP001 In Vivo Characterization

**Figure 3:**
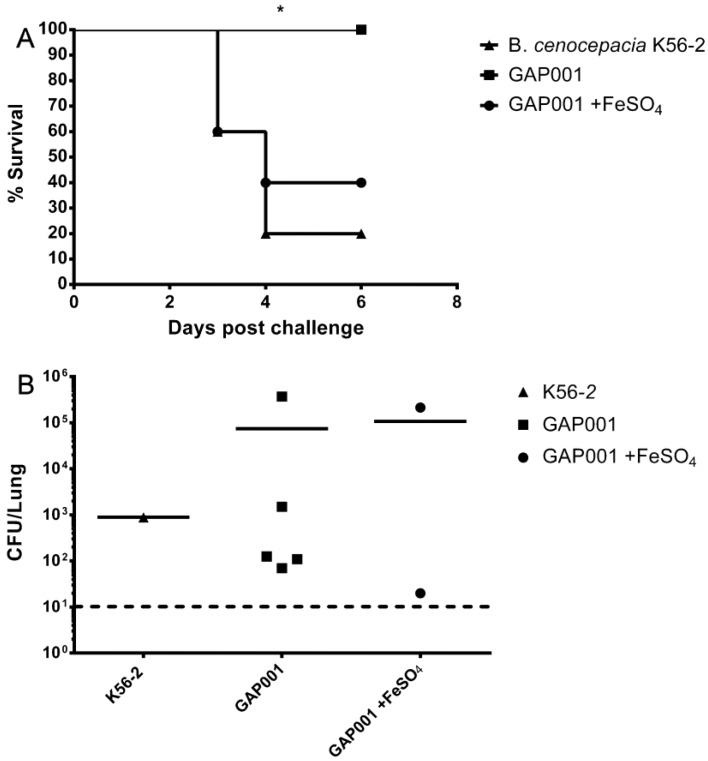
GAP001 is attenuated in BALB/c mice compared to *B. cenocepacia.* GAP001 was unable to kill BALB/c mice at the same dose as *B. cenocepacia*. (**A**) Percent survival of BALB/c mice challenged with 5 × 10^7^ CFU of GAP001 grown in LB (squares) or LB with 200 μM FeSO_4_ (circles) and *B. cenocepacia* K56-2 (triangles). (**B**) Bacterial counts in mice lungs for mice infected with GAP001 grown in LB (squares) or LB with 200 μM FeSO_4_ (circles) and *B. cenocepacia* K56-2 (triangles). Bacterial counts were determined 6 days post infection. The limit of detection was 10 CFU/lung (horizontal dotted line). The Kaplan–Meier method was used and significant differences (*p* ≤ 0.5) were calculated via a log rank test.

Next, we determined whether the GAP001 displayed an attenuated phenotype in vivo. BALB/c mice were infected i.n. with ~5 × 10^7^ CFU of *B. cenocepacia* K56-2 and GAP001 grown with and without iron supplementation. After six days, the totality of mice infected with GAP001 alone (grown in LB) survived, while most mice infected with wild-type (80%) and iron supplemented *tonB* strain (60%) succumbed to the infection (Figure 3A). The lung bacterial load of surviving mice, at 6 dpi (days post-infection) ranged from 70 to 4 × 10^5^ CFU per lung for GAP001-infected mice, 900 CFU for *B. cenocepacia* K56-2, and a range from 20 to 2.1 × 10^5^ for GAP001 supplemented with iron (Figure 3B).

**Figure 4:**
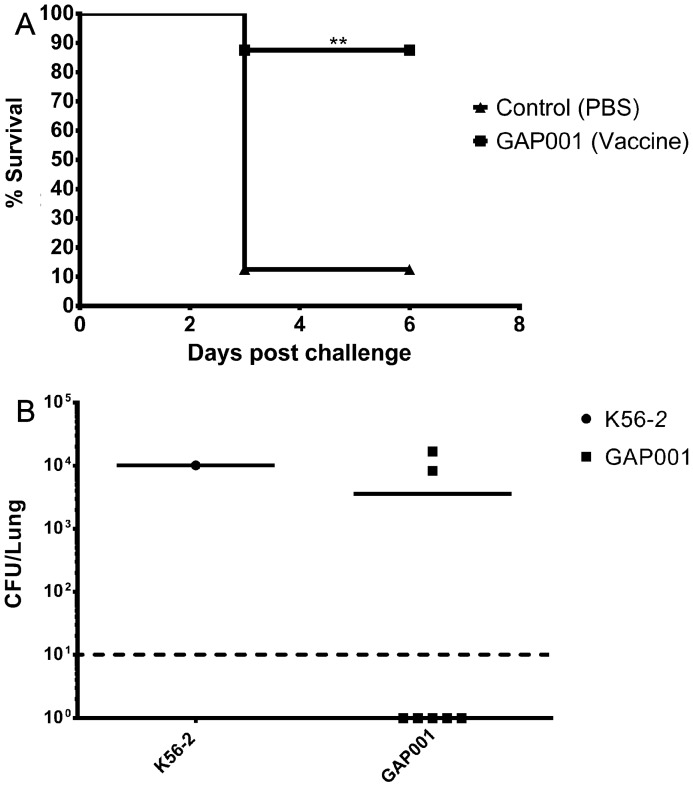
Immunization with GAP001 protects against challenge with *B. cenocepacia.* BALB/c mice immunized with 5 × 10^7^ CFU of GAP001 showed protection against the challenge with *B. cenocepacia* and compared with the control group (PBS). (**A**) Survival curves for the vaccinated (squares) and non-vaccinated (triangles) mice. (**B**) Bacterial loads at 6 days post infection for the vaccinated (squares) and non-vaccinated (triangles) mice. The limit of detection was 10 CFU/lung (horizontal dotted line). The Kaplan–Meier method was used and significant differences (*p* ≤ 0.05) were calculated via a log rank test.

After obtaining 100% in vivo survival with the mutant strain, we tested whether this attenuated strain could be used as a potential vaccine candidate and grant protection to BALB/c mice against a lethal dose of *B. cenocepacia* K56-2 (a nalidixic acid variant of K56-2, Nac^R^, was used to easily differentiate from any potential remaining GAP001 in the mice lungs at the end of the study). Mice received a prime and one boost vaccination scheme, two weeks apart, with 5 × 10^7^ CFU of GAP001. Fourteen days after the last boost, the mice were challenged with 5 × 10^7^ CFU of *B. cenocepacia* K56-2 Nac^R^. Most of the PBS-treated mice (87.5%) succumbed to infection by day three post-challenge, while the animals vaccinated with the *tonB* mutant showed a survival rate of 87.5% at six days post challenge (Figure 4A). Serum was collected 10 days after the boost from vaccinated and PBS control mice (n = 3) and *B. cenocepacia*-specific antibody response was determined. Total IgG presented a mid-high reciprocal end-point titer of 12,800 ± 0, while the control groups showed no serum reactivity (Table 3). The subtypes IgG2a- and IgG1-response to *B. cenocepacia* was also determined, showing a reciprocal end-point titer of 7822 ± 3955 towards IgG2a and levels under the detection limit for IgG1 (Table 4).

To evaluate whether GAP001 immunization granted some degree of sterile immunity, the challenge-surviving BALB/c mice were euthanized and their lungs harvested to determine bacterial colonization. At six days post challenge, 71.4% (five animals) of the surviving GAP001-vaccinated mice cleared bacteria from their lungs (Figure 4B), and presented bacterial loads under the limit of detection (10 CFU). However, 28.6% of the vaccinated mice (two animals) presented lung bacterial counts ~10^4^, similar at the counts observed in the lungs of the only surviving mice from the control group, a 1000-fold reduction from the dose used.

## 4. Discussion

To date, few potential vaccines against Bcc have been developed and studied, and no immune correlates of protection against disease have been clearly defined. Because the use of live attenuated strains usually results in a customized protective response, as well as generating immune memory for lasting protection against defined pathogens, we decided to use an attenuated *B. cenocepacia tonB* strain, since it can provide useful information about the host immune responses required to achieve full protection and eventually, eradication of the pathogen.

After mutant construction, the resulting GAP001 strain showed phenotypes suggesting an inability to uptake bound-iron, as observed in the reduced growth rate in iron-limited conditions and the increased release of siderophores, as the bacteria tries to sequester iron from the environment. However, this mutation does not affect the bacteria’s ability to use free iron, growing at a rate comparable to the wild-type strain and reducing the siderophore release to the media when free iron is supplemented to the media. All these findings are consistent with the results from other *tonB* mutants [14,19], and confirmed the role of TonB in iron transport and the impact of its function on *B. cenocepacia* fitness.

Examination of the mutant strain pathogenicity in vitro through a macrophage uptake assay suggested that the *tonB* mutation affected the bacterial fitness considerably, hindering their survival and showing a clear attenuated phenotype, which could barely be rescued by iron supplementation in the first stages of uptake, causing no relevant effects in the overall survival at the end of the infection.

The strain attenuation was also evident in vivo, as observed in the survival and vaccination studies, in which the mutant had reduced lethality. Similar to the *B. mallei tonB* mutant [14], GAP001 presented an iron-dependent phenotype reversion, in which iron supplementation partially restored the virulence phenotype. These results were not completely unexpected because in order to have a lethal infection model for *B. cenocepacia*, we used an acute infection in which the animals usually succumbed at 3 dpi upon wild-type challenge and, therefore, the attenuated GAP001 only had an increase virulence within the time frame of the study due to the exogenous supplementation of FeSO_4_. 

In contrast to our prior study [14], in which the post-challenge period lasted longer and we observed that the *B. mallei tonB* mutant persisted in the spleen and in some occasions in the lungs of infected animals, our *B. cenocepacia tonB* seemed to be cleared from the lungs of infected animals at a faster rate and very low levels were detected.

The vaccination study showed that GAP001 immunization resulted in a mid- to high-total *B. cenocepacia*-specific IgG and Th1-driven immune response (IgG2a predominant response). Those prior few Bcc related vaccine studies (usually performed with subunit vaccines), have showed different types of immune responses, either Th1 or mixed Th1/Th2 bias [10,11,12], depending mainly on the formulation and/or type of protein used. However, a prior study also suggested that in the case of *B. cenocepacia* infections, a Th1 bias may be needed to effectively diminish the bacterial burden [28], which is consistent with our results. Further, the immune response elicited seems to be effective in clearing bacteria from the infected mice because in most of the animals (71%), the GAP001 vaccination resulted in almost sterilized immunity. These results are also consistent with vaccine studies in the related species *B. mallei,* in which we showed a relationship between a vaccine’s ability to generate specific IgG and Th1-driven immune responses, and the complete protection conferred by the attenuated vaccine against a lethal *B. mallei* challenge [21].

A vaccine that triggers a Th1 response against *B. cenocepacia* could be particularly useful in a CF context, since these patients present an immune response that appears to be tilted towards a Th2 bias [29,30,31,32]. In contrast, given that a Th1 response may be important for the clearance of CF-related pathogens—as observed in CF patients in which Th1 responses have been associated with an overall better pulmonary outcome [30,33], as well as with stable pulmonary infections [34]—a better understanding of this immune response can result in a better outcome. This is also supported by studies in murine models in which *cftr*-deficient mice infected with *P. aeruginosa* showed a recovery from infection associated with a Th1 response [35]. Hence, eliciting a Th1 response could be advantageous to achieve a more balanced Th1/Th2 immune response in the CF lung.

Given the level of protection observed in this study and the antibody titers detected, we consider there is still significant room for vaccine optimization, for example adding an adjuvant to improve the immune response and/or testing alternative vaccination routes. Some insights from the latter could be taken from the intraperitoneal infection models already described for *B. cenocepacia* [28], as well as the development and use of a live attenuated vaccine in the related species *B. pseudomallei*, which has resulted in partial protection against i.n. challenge to a lethal infection [36]. We understand that development of a live attenuated vaccine for CF patients colonized by *B. cenocepacia* or other *Burkholderia* species might be counter-indicated due to the immunocompromised state of these patients; however, the information that we can learn using this attenuated vaccine is extremely valuable in order to further optimize current vaccine candidates or to develop alternative vaccines that can induce a fully protective response.

## 5. Conclusions

In summary, our main goal was to generate and test a *B. cenocepacia* live attenuated vaccine, and the resulting GAP001 strain represents the first *B. cenocepacia* vaccine candidate that grants a high degree of protective immunity with a strong Th1 response. The current results and those generated with the *B. mallei tonB* vaccine [14,21] indicate that a common mutagenesis approach targeting the *tonB* gene has the potential to produce effective vaccine candidates with a potential for cross-protection against other Bcc species, such as *B. multivorans*, as well the other pathogenic *Burkholderia* species such as *B. pseudomallei*. As stated, this study represents an advancement in vaccine development against Bcc infections, as well as providing a platform for further understanding immune responses that can be applicable to a broader array of *Burkholderia* infections.

## Figures and Tables

**Table 1:** Bacterial strains used in this study.

Strain	Relevant Features ^a^	Reference or Source
*B. cenocepacia* K56-2	Human clinical isolate; Tet^s^ Pb^r^	[22]
GAP001	*B. cenocepacia* K56-2 Δ*tonB*	This study
*B. cenocepacia* K56-2 ^b^	Human clinical isolate; Nac^r^ Pb^r^	This study
*E. coli* S17-1 (pGPTcR)	*E. coli* strain containing pGPTcR (pMO130 *tet*) Pb^s^ Tet^r^	This study
*E. coli* S17-1 (pGPTcR-tonB)	Donor strain containing pGPTcR-tonB plasmid; Pb^s^ Tet^r^	This study

^a^ Tet, tetracycline; Pb, polymyxin; ^b^ Nac, Nalidixic acid.

**Table 2:** CAS ^a^ assay halos (mm).

Strain	LB	LB + Fe
*B. cenocepacia* K56-2	22.00 ± 0.36	21.67 ± 0.49
GAP001 ^b^	41.00 ± 1.26 ****	36.17 ± 0.60 ****

**^a^** 10 μL of overnight cultures grown with and without 200 μm FeSO_4_, were spotted in CAS agar plates, the diameter of the halo (mm) formed was determined by duplicates in tree independent experiments. ^b^ *p* ≤ 0.0001 significance (****) in an unpaired *t*-test.

**Table 3:** Iron utilization assay ^a^.

Strain	FeSO_4_	Hemoglobin	Hemin	Lactoferrin	Transferrin
*B. cenocepacia* K56-2 ^b^	+	+	+	+	+
GAP001 ^c^	+	−	−	−	−

**^a^** Overnight cultures were diluted to 1 × 10^5^ CFU/mL in LB + 2,2’-dipyridyl, poured onto plates and then the indicated iron sources were spotted on the plate and growth was assessed. ^b^ 50 μM of 2,2’-dipyridyl were used. ^c^ 300 μM of 2,2’-dipyridyl were used.

**Table 4:** Serum antibody response of BALB/c mice i.n. vaccinated with *B. cenocepacia* live attenuated strain.

Treatment ^a^	Serum titer ^b^ IgG	IgG2a	IgG1
PBS	ND	ND	ND
GAP001/5.4 × 10^7^ CFU	51,200 ± 0	8533 ± 3200	ND

**^a^** Antibody titers were determined at 10 weeks after the boost. PBS control animals were vaccinated with 50 μl of PBS. ^b^ To determine serum antibody titers, sera from 3 mice/group were tested by indirect ELISA with heat-inactivated *B. cenocepacia* K56-2 Nac^R^ whole cells used as the antigen. Titer determinations were performed in triplicate, and data are reported as the mean reciprocal endpoint titers standard deviations (SD). ND, not detected, because titers less than or equal to 100 were considered to represent negative results.

## References

[1] Coenye T., Vandamme P. (2003). Diversity and significance of *Burkholderia* species occupying diverse ecological niches. Environ. Microbiol..

[2] Vandamme P., Dawyndt P. (2011). Classification and identification of the *Burkholderia cepacia* complex: Past, present and future. Syst. Appl. Microbiol..

[3] LiPuma J.J. (2015). Assessing Airway Microbiota in Cystic Fibrosis: What More Should Be Done?. J. Clin. Microbiol..

[4] Pradenas G.A., Ross B.N., Torres A.G. (2016). *Burkholderia cepacia* Complex Vaccines: Where Do We Go from here?. Vaccines (Basel).

[5] Courtney J.M., Dunbar K.E., McDowell A., Moore J.E., Warke T.J., Stevenson M., Elborn J.S. (2004). Clinical outcome of *Burkholderia cepacia* complex infection in cystic fibrosis adults. J. Cyst. Fibros..

[6] Loutet S.A., Valvano M.A. (2010). A decade of *Burkholderia cenocepacia* virulence determinant research. Infect. Immun..

[7] Ganesan S., Sajjan U.S. (2011). Host evasion by *Burkholderia cenocepacia*. Front. Cell Infect. Microbiol..

[8] Saldias M.S., Valvano M.A. (2009). Interactions of Burkholderia cenocepacia and other *Burkholderia cepacia* complex bacteria with epithelial and phagocytic cells. Microbiology.

[9] Lewis E.R., Torres A.G. (2016). The art of persistence-the secrets to *Burkholderia* chronic infections. Pathog. Dis..

[10] Bertot G.M., Restelli M.A., Galanternik L., Urey R.C.A., Valvano M.A., Grinstein S. (2007). Nasal immunization with *Burkholderia multivorans* outer membrane proteins and the mucosal adjuvant adamantylamide dipeptide confers efficient protection against experimental lung infections with *B. multivorans* and *B. cenocepacia*. Infect. Immun..

[11] McClean S., Healy M.E., Collins C., Carberry S., O’Shaughnessy L., Dennehy R., Adams Á., Kennelly H., Corbett J.M., Carty F. (2016). Linocin and OmpW Are Involved in Attachment of the Cystic Fibrosis-Associated Pathogen *Burkholderia cepacia* Complex to Lung Epithelial Cells and Protect Mice against Infection. Infect. Immun..

[12] Makidon P.E., Knowlton J., Groom J.V., Blanco L.P., LiPuma J.J., Bielinska A.U., Baker J.R. (2010). Induction of immune response to the 17 kDa OMPA *Burkholderia cenocepacia* polypeptide and protection against pulmonary infection in mice after nasal vaccination with an OMP nanoemulsion-based vaccine. Med. Microbiol. Immunol..

[13] Hatcher C.L., Muruato L.A., Torres A.G. (2015). Recent Advances in *Burkholderia mallei* and *B. pseudomallei* Research. Curr. Trop. Med. Rep..

[14] Mott T.M., Vijayakumar S., Sbrana E., Endsley J.J., Torres A.G. (2015). Characterization of the *Burkholderia mallei tonB* mutant and Its potential as a backbone strain for vaccine development. PLoS Negl. Trop. Dis..

[15] Skaar E.P. (2010). The battle for iron between bacterial pathogens and their vertebrate hosts. PLoS Pathog..

[16] Mira N.P., Madeira A., Moreira A.S., Coutinho C.P., Sá-Correia I. (2011). Genomic expression analysis reveals strategies of *Burkholderia cenocepacia* to adapt to cystic fibrosis patients’ airways and antimicrobial therapy. PLoS ONE.

[17] Visser M.B., Majumdar S., Hani E., Sokol P.A. (2004). Importance of the ornibactin and pyochelin siderophore transport systems in *Burkholderia cenocepacia* lung infections. Infect. Immun..

[18] Tuanyok A., Kim H.S., Nierman W.C., Yu Y., Dunbar J., Moore R.A., Baker P., Tom M., Ling J.M., Woods D.E. (2005). Genome-wide expression analysis of iron regulation in *Burkholderia pseudomallei* and *Burkholderia mallei* using DNA microarrays. FEMS Microbiol. Lett..

[19] Alvarez B., Alvarez J., Menéndez A., Guijarro J.A. (2008). A mutant in one of two *exbD* loci of a TonB system in *Flavobacterium psychrophilum* shows attenuated virulence and confers protection against cold water disease. Microbiology.

[20] Hsieh P.F., Lin T.L., Lee C.Z., Tsai S.F., Wang J.T. (2008). Serum-induced iron-acquisition systems and TonB contribute to virulence in *Klebsiella pneumoniae* causing primary pyogenic liver abscess. J. Infect. Dis..

[21] Hatcher C.L., Mott T.M., Muruato L.A., Sbrana E., Torres A.G. (2016). *Burkholderia mallei* CLH001 Attenuated Vaccine Strain Is Immunogenic and Protects against Acute Respiratory Glanders. Infect. Immun..

[22] Mahenthiralingam E., Coenye T., Chung J.W., Speert D.P., Govan J.R., Taylor P., Vandamme P. (2000). Diagnostically and experimentally useful panel of strains from the *Burkholderia cepacia* complex. J. Clin. Microbiol..

[23] Torres A.G., Payne S.M. (1997). Haem iron-transport system in enterohaemorrhagic *Escherichia coli* O157:H7. Mol. Microbiol..

[24] Louden B.C., Haarmann D., Lynne A.M. (2011). Use of Blue Agar CAS Assay for Siderophore Detection. J. Microbiol. Biol. Educ..

[25] Lewis E.R.G., Kilgore P.B., Mott T.M., Pradenas G.A., Torres A.G. (2017). Comparing *in vitro* and *in vivo* virulence phenotypes of *Burkholderia pseudomallei* type G strains. PLoS ONE.

[26] Hamad M.A., Zajdowicz S.L., Holmes R.K., Voskuil M.I. (2009). An allelic exchange system for compliant genetic manipulation of the select agents *Burkholderia pseudomallei* and *Burkholderia mallei*. Gene.

[27] Gauthier G.M., Sullivan T.D., Gallardo S.S., Brandhorst T.T., Vanden Wymelenberg A.J., Cuomo C.A., Suen G., Currie C.R., Klein B.S. (2010). SREB, a GATA transcription factor that directs disparate fates in *Blastomyces dermatitidis* including morphogenesis and siderophore biosynthesis. PLoS Pathog..

[28] Speert D.P., Steen B., Halsey K., Kwan E. (1999). A murine model for infection with *Burkholderia cepacia* with sustained persistence in the spleen. Infect. Immun..

[29] Tiringer K., Treis A., Fucik P., Gona M., Gruber S., Renner S., Dehlink E., Nachbaur E., Horak F., Jaksch P. (2013). A Th17- and Th2-skewed cytokine profile in cystic fibrosis lungs represents a potential risk factor for *Pseudomonas aeruginosa* infection. Am. J. Respir. Crit. Care Med..

[30] Moser C., Kjaergaard S., Pressler T., Kharazmi A., Koch C., Høiby N. (2000). The immune response to chronic *Pseudomonas aeruginosa* lung infection in cystic fibrosis patients is predominantly of the Th2 type. APMIS.

[31] Moser C., Jensen P.Ø., Pressler T., Frederiksen B., Lanng S., Kharazmi A., Koch C., Høiby N. (2005). Serum concentrations of GM-CSF and G-CSF correlate with the Th1/Th2 cytokine response in cystic fibrosis patients with chronic *Pseudomonas aeruginosa* lung infection. APMIS.

[32] Hartl D. (2009). Immunological mechanisms behind the cystic fibrosis-ABPA link. Med. Mycol..

[33] Hartl D., Griese M., Kappler M., Zissel G., Reinhardt D., Rebhan C., Schendel D.J., Krauss-Etschmann S. (2006). Pulmonary T(H)2 response in *Pseudomonas aeruginosa*-infected patients with cystic fibrosis. J. Allergy. Clin. Immunol..

[34] Wojnarowski C., Frischer T., Hofbauer E., Grabner C., Mosgoeller W., Eichler I., Ziesche R. (1999). Cytokine expression in bronchial biopsies of cystic fibrosis patients with and without acute exacerbation. Eur. Respir. J..

[35] Moser C., Jensen P.O., Kobayashi O., Hougen H.P., Song Z., Rygaard J., Kharazmi A., Høiby N. (2002). Improved outcome of chronic *Pseudomonas aeruginosa* lung infection is associated with induction of a Th1-dominated cytokine response. Clin. Exp. Immunol..

[36] Silva E.B., Dow S.W. (2013). Development of *Burkholderia mallei* and *pseudomallei* vaccines. Front. Cell Infect. Microbiol..

